# Pharmacological Interventions in the Management of Antipsychotic-Induced Metabolic Disturbances

**DOI:** 10.1192/j.eurpsy.2025.2064

**Published:** 2025-08-26

**Authors:** J. Barreira, S. Macedo

**Affiliations:** 1 Psychiatry and Mental Health, Unidade Local de Saúde do Tâmega e Sousa, Porto, Portugal

## Abstract

**Introduction:**

Antipsychotic medications, particularly atypical antipsychotics, are commonly associated with metabolic side effects such as weight gain, dyslipidemia, and insulin resistance. These disturbances significantly increase the risk of cardiovascular disease and mortality, especially in patients treated with clozapine and olanzapine. It is not always feasible to discontinue these treatments, as the decision largely depends on the clinical context. Therefore, addressing these metabolic side effects requires specific pharmacological interventions to mitigate their impact.

**Objectives:**

This non-systematic review aims to assess the evidence supporting pharmacological interventions in managing antipsychotic-induced metabolic disturbances.

**Methods:**

Relevant and recent studies or reviews were selected from the PubMed electronic database using search terms related to antipsychotic-induced metabolic disturbances and pharmacological interventions to manage them.

**Results:**

Current evidence suggests the need for early and aggressive pharmacological intervention in patients experiencing antipsychotic-induced weight gain. Non-pharmacological interventions, such as physical activity and dietary changes, are often insufficient to mitigate these iatrogenic effects. Pharmacological interventions to reduce metabolic risk in individuals with severe mental illness may include the introduction of an antipsychotic with a more favourable metabolic profile, modification of antipsychotic therapy (dose adjustment, augmentation with another antipsychotic with a lower metabolic risk or switching to another antipsychotic with a lower metabolic risk) and treatment of medical conditions (through the use of drugs such as metformin, statins, among others). Based on updated scientific evidence, the most effective pharmacological treatments for reducing weight gain associated with second-generation antipsychotics are metformin, GLP-1 receptor agonists, topiramate, zonisamide, and nizatidine. The adjunctive use of aripiprazole also reduces lipid levels and weight and attenuates negative symptoms in patients with schizophrenia and metabolic syndrome. Metformin is considered the best-tolerated intervention, while topiramate is the least tolerated.

**Conclusions:**

Pharmacological interventions, particularly the use of metformin and GLP-1 analogues, offer promising results in managing antipsychotic-induced metabolic disturbances. These interventions improve weight management, glucose levels, and lipid profiles. More large-scale randomized trials are needed to further validate these interventions and assess long-term safety and efficacy.

**Disclosure of Interest:**

None Declared

